# Artificial intelligence (AI) in medicine, current applications and future role with special emphasis on its potential and promise in pathology: present and future impact, obstacles including costs and acceptance among pathologists, practical and philosophical considerations. A comprehensive review

**DOI:** 10.1186/s13000-021-01085-4

**Published:** 2021-03-17

**Authors:** Zubair Ahmad, Shabina Rahim, Maha Zubair, Jamshid Abdul-Ghafar

**Affiliations:** 1 Department of Pathology and Laboratory Medicine, Aga Khan University Hospital, Karachi, Pakistan; 2 Department of Pathology and Clinical Laboratory, French Medical Institute for Mothers and Children (FMIC), Kabul, Afghanistan

**Keywords:** Artificial intelligence, Medicine, Pathology

## Abstract

**Background:**

The role of Artificial intelligence (AI) which is defined as the ability of computers to perform tasks that normally require human intelligence is constantly expanding. Medicine was slow to embrace AI. However, the role of AI in medicine is rapidly expanding and promises to revolutionize patient care in the coming years. In addition, it has the ability to democratize high level medical care and make it accessible to all parts of the world.

**Main text:**

Among specialties of medicine, some like radiology were relatively quick to adopt AI whereas others especially pathology (and surgical pathology in particular) are only just beginning to utilize AI. AI promises to play a major role in accurate diagnosis, prognosis and treatment of cancers. In this paper, the general principles of AI are defined first followed by a detailed discussion of its current role in medicine. In the second half of this comprehensive review, the current and future role of AI in surgical pathology is discussed in detail including an account of the practical difficulties involved and the fear of pathologists of being replaced by computer algorithms. A number of recent studies which demonstrate the usefulness of AI in the practice of surgical pathology are highlighted.

**Conclusion:**

AI has the potential to transform the practice of surgical pathology by ensuring rapid and accurate results and enabling pathologists to focus on higher level diagnostic and consultative tasks such as integrating molecular, morphologic and clinical information to make accurate diagnosis in difficult cases, determine prognosis objectively and in this way contribute to personalized care.

## Introduction

The role of Artificial intelligence (AI) in the field of medicine is constantly expanding. AI promises to revolutionize patient care in the coming years with the aim of optimizing personalized medicine and tailoring it to the use of individual patients. Medicine embraced AI slowly. Some specialties such as radiology were quick to adopt AI. Others like pathology are only now beginning to utilize AI in clinical use. In this article, we will start by describing the general principles of AI. We will then analyze its current role in medicine in general by looking at various examples of its applications in specialties such as radiology and oncology. In the second half of this review, we will discuss the current and expected future role of AI in surgical pathology, the practical, financial and regulatory difficulties involved, the future of the microscope and the fear of pathologists of being replaced by computer algorithms. We will also discuss a number of recent studies which highlight the usefulness of AI in the practice of surgical pathology.

## Main text

### General principles and definitions

AI can be defined as the ability of computers to perform tasks that normally require human intelligence. It refers to the development and programming of computers and devices having human-like characteristics which can perform diverse complex functions such as driving an automatic car, diagnosing a medical condition, discovering fraud and money laundering, interpreting legal advice, proving mathematical theorems etc.

### Machine learning (ML)

a subfield of AI, is defined as a computational system based on a set of algorithms that attempts to analyze vast and diverse data by using multiple layers of analysis. There are a number of ways in which a computer can be programmed to make intelligent judgments and it is essential to use the right algorithms for specific purposes. ML is one of the commonest AI techniques used for processing big data. It is a self-adaptive system that provides increasingly better analysis and patterns with experience and newly added data. These techniques have evolved hand – in – hand with the digital era which has brought about an explosion of data in all forms from all parts of the world. Enormous amount of data, known simply as big data is easily and readily accessible and can be shared through applications like cloud computing.

ML applies statistical methods to automatically learn from data and experience without explicit instructions. It has seen an explosion of interest in recent years. One technique in particular, known as deep learning, has produced ground breaking results in many important problems including image classification and speed recognition.

Recent growing interest and efforts to incorporate AI into any number of industries is mainly due to the rise of deep learning. Also known as deep neural learning or deep neural network or convoluted neural network (CNN), it is a type of machine learning algorithm that uses multiple layers of data for example multiple layers of image processing to access higher level features from the image. It is a function of AI which is inspired by the neurons of the human brain and imitates the functioning of the human brain in processing data and creating patterns which can be used in decision making. It is capable of learning unsupervised from data. Deep or convoluted neural networks are the most used ML techniques in the biomedical world. These artificial neural networks are interconnected and follow mathematical models. Their field of application is vast and allows the management of ‘big data’ in genomics and molecular biology. They are most commonly applied for analyzing visual images.

Through deep learning, AI recognizes patterns using various forms of neural networks based on the availability of big data repositories. The process is inexpensive and the computational processing power is easily accessible. The neural networks can acquire data rapidly via image scanners, digital cameras, remote sensors, electronic appliances or the Internet of Things (IoT). AI has the ability to learn from both unstructured and unlabeled data. It uses a hierarchical level of artificial neural networks to carry out the process of ML. Artificial neural networks used in AI are built, as mentioned above, like the human brain with neuron nodes connected together like a web. This is in contrast with traditional computer programs which build analysis with data in a linear way. The hierarchical design of deep learning systems enables machines to process data via a nonlinear approach [1].

Big data is usually unstructured and is so vast that it could take years, even decades for humans to understand it, process it and obtain relevant information from it. The unaided human brain cannot extract meaning from such vast data sets. Thus, it is necessary to use computers to identify patterns and associations and make inferences and predictions from the data. Companies in all spheres and fields have now realized the incredible potential of unraveling this wealth of information and are increasingly adapting to AI systems. Exponential growth in computing power, data storage and sensing technology is producing a new world in which incredible amount of data can be captured and analyzed.

Owing to the breakthrough of deep learning, AI rapidly developed in the 2010s. Utilizing highly advanced computer processing power and software technology, AI is expected to radically change our lives, industries and society as a whole. It has now entered the era of full- scale practical dissemination.

### AI in medicine, the present and the future

Advances in ML algorithms are resulting in the replication of many medical tasks which currently require human expertise by AI systems at levels of accuracy similar to or greater than that achieved by human experts. In medicine**,** deep learning applications are increasingly being trained with large amounts of annotated data sets freeing medical specialists to focus on more productive tasks and projects. The potential of AI in medicine is limitless and can serve as a great boon to improve health care delivery in clinical practice.

According to Goldenberg et al., computer based decision support systems based on ML have the potential to revolutionize medicine by performing complex tasks that are currently assigned to specialists. ML systems can increase diagnostic accuracy, increase efficiency of thorough puts, better streamline clinical workflow, decrease human resource costs and improve treatment choices [2].

However effective use of AI in medicine requires synergistic transdisciplinary competencies. In medicine, recent promising biomedical and biomarker discoveries notwithstanding, individually tailored care is still far from reality and novel therapies which emerge from preclinical trials are very rarely translatable to evaluation for their diagnostic and therapeutic potential. The discrepancy between experimental data on new anticancer molecules and their actual use in diagnosis and therapy is due to a number of factors which include biological differences between human disease and animal models, inconsistencies in experimental methodologies, wrong interpretation of experimental results, lack of validation of such data by pathologists with long term experience in animal cancer models etc. For example, personalized care in oncology requires the synergistic combination of several disciplines such as nuclear medicine, radiology and surgical pathology which represent complementary approaches to diagnosis, prognosis and evaluation of therapeutic response. A structural collaboration model between these disciplines can accelerate the achievement of a medical paradigm which takes into consideration the uniqueness of every human being. A recent study examined fifteen papers focusing on early and accurate diagnosis of breast, lung and prostate cancer and lymphoma using innovative AI applications. One example was the application of a deep neural network in discriminating between malignant breast cancer lesions in mammographic images. The authors of this study were confident that the data they examined provides the scientific rationale for further investigations in translational medicine based on the combination between surgical pathology, radiology and nuclear medicine [3].

ML is pioneering a new paradigm for scientific research. Traditionally, the classic ‘hypothesis testing’ approach is used in research in which processing of data leads to explanatory mechanisms which then suggest further experiments that in turn lead to classic findings. As a result of rapid technological advances, however, many experiments now collect vast amounts of information and can be considered ‘hypothesis generating’. Investigations in genomics and other-omics are cases in point. The advent of image digitization has led to experiments which generate enormous gigabytes or terabytes of data. Fortunately, advances in deep learning allow the derivation of important qualitative and quantitative information from images, putting visual observation on the same playing field as molecular analysis. Since deep learning is also very useful for- omic analysis, it allows the amalgamation and interpretation of image based data with – omic information, allowing this data to be used for providing new and more accurate knowledge. In this new hypothesis generating paradigm, we hunt for meaning in a huge data set instead of proceeding one logical step at a time from observation to better explanations. Thus deep learning has turned the scientific process on its end [4].

AI has already become a major element in the health care landscape. It has already become a reality providing value in many fields of medicine for example in assessment of skin lesions, evaluating fundus retinography for detection of diabetic retinopathy, radiologic diagnosis for example interpretation chest radiographs etc. These examples highlight the value of AI in aiding clinicians to improve quality, safety, diagnosis and democratization of care. For example, AI has enable radiologists to read imaging studies from anywhere in the world at their own institution bringing expert care to parts of the world where it is not available. In using AI for better medical care the investment of time and manpower to validate model data sets is a major hurdle. Because Deep learning AI identifies patterns, the data used to train the AI model must be validated by medical specialists. Additional financial resources and considerable time are required to perfect AI models which can be deployed with confidence to assist in medical practice.

The question of legal responsibility will need to be resolved before AI can become common place in medicine especially in imaging specialties such as radiology and pathology. Who will be held accountable for an action resulting from an AI based decision? Who will be responsible for an error made through the use of an AI program? It cannot be emphasized enough that legal responsibility is a significant consideration in the medical field when implementing new technologies or procedures or when developing new drugs. Use of AI is new in medicine and no legal precedents are available. No matter how much more accurate AI tools are compared to their human counterparts, the possibility that data might be misinterpreted through false positive and false negative findings cannot be excluded. The issue of legal responsibility for AI based decisions in medicine is further complicated and obscured by the lack of clarity and confusion regarding major issues such as processing of sensitive personal information and data collection, consent, transparency, storage etc. The human element will remain an important factor in incorporating AI into wide practice in hospitals. In pathology, even if the process is completely automated with a routine digital imaging system and data base management, human agreement will likely be essential [5].

Although health care was slow to adopt AI, the pace of implementation is now accelerating at an impressive rate. In 2014, the acquisition of AI startups in health care was about 600 million dollars. In 2021, it is anticipated to reach 6.6 billion dollars. One reason health care is ripe for AI is “big data”. The health care industry has rich data sets which are ideal for AI [6]. Personalized care is the major objective of both basic and translational cancer research. Building an intelligent automated entity to evaluate, diagnose and treat patients in research settings is arguably the easiest part of designing an end –to- end medical AI system. There is a lot of hype and hopes surrounding emerging AI applications in medicine but the brittleness of these systems, the importance of defining the correct frameworks for their application and the need to ensure vigorous quality control including human supervision to avoid driving patients on ‘autopilot’ towards unexpected, unwanted and unhealthy outcomes are essential factors that need to be acknowledged. Since modern machine learning algorithms perform complex mathematical transformations to the input data, errors made by computational systems will require extra vigilance for detection and interpretation [7].

AI will in all probability transform clinical practice over the next decade. As AI building an intelligent automated entity to evaluate, diagnose and treat patients in research settings is arguably the easiest part of designing an end –to- end medical AI system. There is a lot of hype and hopes surrounding emerging AI applications in medicine but the brittleness of these systems, the importance of defining the correct frameworks for their application and the need to ensure vigorous quality control including human supervision to avoid driving patients on ‘autopilot’ towards unexpected, unwanted and unhealthy outcomes are essential factors that need to be acknowledged. Since modern machine learning algorithms perform complex mathematical transformations to the input data, errors made by computational systems will require extra vigilance for detection and interpretation [7].

AI will in all probability transform clinical practice over the next decade. As AI technologies are evolving at a fast pace and machine learning models are being updated with additional pieces of information, regulatory approval is essential. Recently, the Food and Drug Administration (FDA) announced a pilot certification approach that inspects both the AI developers and the product itself [8]. Such steps can ensure public trust in novel medical AI applications. Even if an AI system is designed only to advise physicians or health care personnel rather than to carry out the actual diagnosis and treatment tasks, it may still result in unintended harmful consequences. A recent study showed that over-reliance on decision support systems resulted in increased false negative rate in radiology diagnoses compared with the study scenario when the computer-aided diagnostic system was unavailable to the same group of radiologists- this is termed confirmatory bias. In addition, inexperienced medical practitioners may overreact to excessive warning messages - this is termed alert fatigue. Thus, AI developers need to address these challenges even if the systems only play an advisory role.

Trust in medical technology is closely related to its anticipated utility. If a perception emerges that a new technology is harmful and has untoward consequences, then the barriers to its acceptance will become next to insurmountable. This issue becomes even more complicated if the technology is complex and the general public and even the domain expert cannot fully evaluate its efficacy and potential hazards. This is termed ‘Frame Problem’. Frame problem can cause medical errors that will draw the attention of the public and lead to law suits against companies or organizations using, deploying or developing medical AI applications. The ‘black box’ nature (lack of full disclosure and information about the technology) of modern machine learning algorithms may further exacerbate and aggravate the issue. High profile examples of harmful or inadequate performance will result in extra scrutiny of the whole field and may hinder the development of more robust AI systems. It must be kept in mind that data driven AI algorithms are not immune from the ‘garbage - in garbage – out’ rule. ML algorithms are designed to identify the hidden patterns of the data and generate output projections based on what they have seen in the past. As many input data sets contain artifacts or biases, the models learnt from such data carry those biases and can potentially amplify them with harmful consequences for the patients. Thus, optimized machine learning models in medicine can be confounded by their training data, may not reflect an objective clinical assessment and lead to partiality and mistakes. Thus, great attention needs to be paid to data quality and provenance in order to foster ‘patient trust’ in AI systems and to avoid unethical practices even if only due to negligence. However, this is an expensive task. In other words, since modern machine learning algorithms make complex mathematical changes to the input data, biases and errors made by computational systems will require extra vigilance to detect and interpret [9].

Let us now examine various examples of the application of AI in various subspecialties of medicine including gastroenterology, ophthalmology, dermatology, surgery, radiology, oncology etc. The promise of AI in health care is the delivery of improved quality and safety of care and the potential to democratize expertise.

Nakagawa et al. developed a deep learning based AI system for assessment of superficial esophageal squamous cell carcinoma (SCC). Invasion of tumor depth is a critical factor which affects the choice of treatment. However, assessment of cancer depth is subjective and inter observer variability is common. The authors obtained 8660 non-magnified endoscopic (non-ME) and 5678 ME images as training data set and 405 non- ME images from 155 patients as validation set. The system showed sensitivity and specificity of 90.1 and 95.8% respectively and its performance in diagnosing depth of invasion in superficial SCC was comparable to that of experienced endoscopists [10].

Similarly, Horie et al. developed a deep learning CNN and tested its ability to diagnose esophageal SCC and adenocarcinoma. They retrospectively collected 8428 training images of known esophageal carcinoma from 384 patients at their hospital and then prepared 1118 test images from 47 patients with esophageal cancer and 50 patients without cancer to evaluate diagnostic accuracy. The CNN took just 27 s to analyze the test images with a sensitivity of 98%. It detected all cancers less than 10 mm in size. The authors were confident that more training would lead to even greater diagnostic accuracy thus facilitating early diagnosis with consequent better prognosis for patients with esophageal cancer [11].

Hirasawa et al. developed a CNN to detect gastric cancer in endoscopic images. They trained their CNN- based diagnostic system using 13,584 endoscopic images of gastric cancer. To evaluate the diagnostic accuracy, an independent test set of 2296 images collected from 69 consecutive patients with 77 gastric cancer lesions was applied to the constructed CNN. The CNN correctly diagnosed 71 of 77 cancer lesions with an overall sensitivity of 92.2%. 70 of the 71 lesions (98.6%) with a diameter of 6 mm or more as well as all invasive cancers were correctly detected. All missed lesions were superficially depressed and differentiated – type intra mucosal cancers that were difficult to distinguish from gastritis even for experienced endoscopists. Thus, the system developed by Hirasawa et al. was able to process numerous stored endoscopic images in a very short time with a clinically relevant diagnostic ability and could be applied to daily clinical practice to reduce the burden of endoscopists [12].

Esteva et al. developed a deep CNN to discriminate between the most common skin cancers including malignant melanoma. They compared their algorithm against 21 board certified dermatologists in evaluating biopsy proven clinical images and demonstrated at least equivalent if not outright superiority. The authors suggested that mobile devices, like smart phones, could be deployed with similar algorithms, permitting potentially low cost universal access to vital diagnostic care anywhere in the world [13]. Digital pathology is becoming the new standard of care in dermatology and personalized medicine. This collaboration between dermatopathology and dermatology (personalized dermatology) is aimed at therapy tailored to the specific needs of each patient [14].

In another recent study, Gulshan et al. applied a deep CNN approach to a test set of more than 128,000 retinal fundus images from adult patients with diabetes to identify referable diabetic retinopathy. The algorithm that they developed demonstrated a very high sensitivity and specificity for detecting referable diabetic retinopathy and macular edema. This study showed that AI will not be used to replace physicians but rather to perform simple, cost effective and widely available examinations and analyses which could help identify at risk patients who require referral for specialty care while reassuring other patients that retinopathy was not present [15].

In a recent study, Dong et al. used machine learning algorithms to predict the disease course of Crohn’s Disease in Chinese patients. Crohn’s disease is a complex disease and it is difficult to predict its course. Their proposed machine learning model accurately predicted the risk of surgical intervention in their patients. The authors were confident that it could be used to design treatment strategies tailored to individual Cohn’s Disease patients [16].

Even in surgery, the same critical question is now being asked. How will the digital revolution and AI change surgical practice? How will it translate in the practice of surgery? A recent study by Wall and Krummel believes that AI may impact surgery in the near future in three main areas. These include enhancement of training modalities, cognitive enhancement of the surgeon and procedural automation. The authors admit that there have been unanticipated missteps in the use of these technologies but have little doubts about their adoption in surgical practice. The authors agree that the promise of big data, AI and automation in surgery is high and believe that surgeons in the near future will need to become “digital surgeons” and must be prepared to adopt smarter training modalities, supervise the learning of machines that can enhance cognitive function and ultimately oversee autonomous surgery without allowing for a decay in their operating skills [17].

### AI in radiology, already a success story

In radiology, AI is improving accuracy in diagnostic imaging. As patient images can be directly acquired in digital form for central archival and soft copy review, radiology practice has readily incorporated AI into clinical practice. An established digital imaging infrastructure has allowed a seamless embedding of AI into the radiology workflow. Large amounts of data can be translated and transmitted within minutes. For patient images generated by different imaging modalities such as X-ray, Magnetic Resonance Imaging (MRI), Computed Tomography (CT), ultrasound, and mammogram, deep learning AI can be automated to pinpoint accurately the areas of interest and diagnosis. Image recognition using AI with deep learning through CNNs has dramatically improved and is being increasingly applied for diagnostic imaging [5].

Radiology converted to digital images more than 25 years ago. It is well positioned to deploy AI for diagnostics. Several studies have shown considerable success in the use of AI for evaluating a variety of scan types including mammography for breast lesions, CT scans for lung nodules and infections and MRI images for brain tumors. By converting to digital images, radiology eliminated film, chemicals, developers and storage of films and solved problems related to loss of films and transport of films to where they are needed for example intensive care units (ICUs), operating rooms (ORs) and emergency departments. There was inherent value within these images for greater learning using computers to improve the quality, safety and efficiency of radiologists. However, the role of radiologist remains crucial. For example, chest x-ray films with atypical features would still need to be reviewed by radiologists to ensure that artifacts or unusual clinical contexts were adequately captured. An AI system will need to be continually calibrated by human feedback.

Lee et al. developed a deep learning based computer-aided diagnosis (CAD) system for use in diagnosis of cervical lymph node metastases by CT scan in thyroid cancer. The authors collected 995 axial CT images (647 benign and 348 malignant) from 202 patients who underwent CT for planning of surgery. Their system was able to classify cervical lymph node metastases with a high degree of accuracy. The authors believe that their model may be useful in the clinical setting for the above purpose [18].

A 2019 radiology based study tested the effect of AI in automatic identification of lung cancer nodules (1 mm and 5 mm thick) by CT in patients with stage T1 lung cancer and compared the results with manual screening of T1 lung cancer nodules by CT. It needs to be understood that using CT to screen lung cancer nodules is a huge workload. The AI recognition technology used in the study learned by computer neural network methods 5000 cases of stage T1 lung cancer patients with 1 mm and 5 mm thickness nodules. Following this learning, 500 cases of chest CT in stage T1 lung cancer patients with 1 mm and 5 mm thickness nodules were tested with AI and results were compared with those of artificial manual reading by radiologists. The detection rates were similar and no significant differences were noted. Thus, automatic learning of early lung cancer chest CT images by AI showed high specificity and sensitivity for early lung cancer identification and may prove invaluable in the near future in assisting doctors in the early diagnosis of small lung cancer nodules [19].

Value of a synergistic approach between disciplines using AI for cancer prognostication and therapy: AI, through the use of convolutional networks is also expected to play an important role in prediction of cancer outcome. Survival outcome is the most important outcome for cancer patients so that they can plan for themselves and their families. Thus, determining cancer progress is crucial to controlling suffering and death due to cancer. As the histologic diagnosis of cancer is an essential initial step in determining the line of therapy, pathologists have a great responsibility in the diagnosis of cancer. Histologic grading and staging systems based on the size or extent of invasion of the primary tumor, involvement of regional lymph nodes and presence or absence of distant metastases are currently used to predict the biologic behavior of cancer. In addition to histology, several other tools including genomic markers, gene expression and epigenetic modifications are also used to predict outcome in cancer patients [20].

Since personalized medicine is the main objective of cancer research, it is essential to form an alliance between imaging diagnostics (radiology and nuclear medicine) and surgical pathology since a structured collaboration model between these disciplines can speed up the achievement of a paradigm for personalized medicine. A recent study presented automatic glioma grade identification from MRI images using deep convolutional neural network. Glioma images were collected from government hospitals and the AI system categorized the tumors into four grades: low grade glioma, oligodendroglioma, anaplastic glioma and glioblastoma multiforme. The results showed reasonably good performance with high classification accuracies [21]. Another recent study by Mobadersany et al. utilized digital pathology images and genomic markers to predict overall survival of brain tumors. The authors examined the ability of AI in predicting overall survival in diffuse gliomas. Histologic grading and genomic classifications have independent prognostic power in such predictions. Until the recent past, histologic diagnosis and grading were used but the recent emergence of molecular subtyping has resolved the uncertainty related to lineage. Criteria for grading gliomas need to be redefined in the context of molecular subtyping. Improving the accuracy and objectivity of glioma grading will directly impact patient care by identifying patients with aggressive disease who require more aggressive therapeutic regimens and sparing those with less aggressive disease from unnecessary treatments. Currently, all grade III and IV diffuse gliomas are typically treated very aggressively with radiation and concomitant chemotherapy. In the above mentioned study, the AI software learned about survival from histologic images and created a unified framework which interpreted histology and genomic biomarkers for predicting time to event outcomes. The ability of predicting patient outcomes by AI was found to be more accurate than that of surgical pathologists. This study provides insights into applications of deep learning in medicine and the integration of histology and genomic data and provides methods for dealing with factors such as intratumoral heterogeneity [22]. Similarly, Muneer et al. used AI techniques for glioma grade identification and their results were excellent with greater than 90% accuracy [23]. The clinical success of Immunotherapy is driving the need to develop new prognostic and predictive assays for patient selection and stratification. This can be achieved by a combination of computational pathology and AI. A recent study critically assessed various computational approaches which can help in the development of a standardized methodology in the assessment of immune oncology biomarkers such as PDL-1. The authors discussed how integrated bio informatics allow the amalgamation of complex morphological phenotypes with AI. They provided an outline of ML and AI tools which can be applied in immuno -oncology for example pattern recognition in large and complex data sets and deep learning approaches for survival analysis. They were hopeful that combinations of surgical pathology and computational analysis will improve patient stratification in immuno – oncology. The authors are convinced that future clinical demands will be best met by dedicated research at the interface of surgical pathology and bio informatics supported by professional societies and by incorporating data sciences and digital image analysis in the professional education of pathologists [24].

### AI in oncology

In Oncology, new AI intelligence platforms could in future assist in making therapeutic decisions in cancer patients [25]. In 2016, the results of a double-blind validation study were presented at the San Antonio Breast Cancer Symposium which demonstrated a strong concordance between treatment recommendations by a panel of oncologists and Watson for oncology (WFO), an AI platform which was developed by IBM Corporation, (Armonk, NY) in collaboration with Memorial Sloan Kettering Cancer Center. WFO computing system has the ability to extract and assess large amounts of structured and unstructured data from medical records. It then uses natural language processing and machine learning to present cancer treatment options. In this study, the authors compared the concordance between WFO and the multidisciplinary tumor board of the institution (a group of 12 to 15 oncologists). The degree of concordance was analyzed in 638 patients with breast cancer who had been treated at the hospital. The time it took for each method to issue recommendations was also analyzed. The study showed that 90% of WFO’s recommendations were concordant with those of the tumor board. Nearly 80% of the recommendations were concordant in patients with non – metastatic breast cancer. However, concordance was only 45% in patients with metastatic disease, 68% in patients with triple negative breast cancer and 35% in patients with HER 2/neu negative breast cancer. The authors noted that patients with triple negative breast cancer have fewer treatment options compared to those with HER 2/neu negative breast cancer. More complicated cancers lead to more divergent opinions regarding treatment. The authors found that it took an average of 20 min to manually capture and analyze the data and generate recommendations. However, after the oncologists gained more familiarity with the cases, the mean time decreased to approximately 12 min. WFO, on the other hand took only 40 s to capture and analyze the data and make recommendations. According to the authors of this study, WFO can provide treatment recommendations not only for patients with breast cancer but also for those with lung and colorectal cancer. The lead author’s institution in India recently adopted the WFO system to support oncologists in making quality, evidence based treatment decisions in cancer patients. The study however cautioned that though AI is a helpful step toward personalized medicine, it can only complement the physician’s work, not replace it. This is because when dealing with humans, many factors such as the context and preference of each patient, the patient-physician relationship, human touch and empathy are present which cannot be addressed by a machine [26].

Liu et al. in 2018 published a feasibility study using WFO to assess its ability to make treatment recommendations in Chinese patients with lung cancer. In the authors’ own words, “WFO is an outstanding representative AI in the medical field, and it can provide to cancer patients prompt treatment recommendations comparable with ones made by expert oncologists”. WFO is increasingly being used in China. The authors selected all lung cancer patients who were hospitalized and received antitumor treatment for the first time at their hospital. They used WFO to make treatment recommendations for their patients and then compared these recommendations to those made by (or treatment regimens administered by) their expert multi-disciplinary team. Almost 66% recommendations made by WFO were consistent with the recommendations made by the oncology experts. They concluded that though WFO recommendations were consistent with the experts in the majority of cases, they were still inconsistent in a significantly high proportion of cases and thus, WFO could not currently replace oncologists. They asserted that WFO can improve the efficiency of clinical work by providing assistance to doctors but it needs to learn the regional, ethnic characteristics of patients to improve its assistive ability [27].

### AI in surgical pathology: its potential and promise, difficulties and obstacles, current applications and future directions

More than a decade ago, a number of articles described in detail the steps by which AI could be applied for routine tissue based diagnosis using ‘virtual slides’ or in other words the presentation of microscopic images as a whole in a digital matrix. These steps include the measurement of individual image quality and its correction if unsatisfactory, development of a pixel based diagnostic algorithm and its application in diagnosis and classification and feedback to order additional information. Examples include virtual Immunohistochemical slides, automated image classification, detection of relevant image information, and supervision by pathologists. These early studies hoped that pathologists will no longer be primary “water carriers” but will work as supervisors at a “higher level” and AI will allow them more time to concentrate on difficult cases for the benefit of their patients. Even in 2009, virtual slides were already in use for teaching and continuous education and first attempts to introduce them into routine work had begun. At that time the implementation of a complete connected AI supported system was in its childhood [28].

Advances in the quality of whole –slide images have set the stage for the clinical use of digital images in surgical pathology. Along with advances in computer image analysis, this raises the possibility for computer assisted diagnostics in pathology to improve histopathologic interpretation and clinical care.

Pathology was late to adopt digital imaging and computer assisted diagnostic technologies. This is partly due to practical and financial obstacles. It needs to be understood that unlike radiology, many of the practical benefits cannot be achieved with pathology digitization. A surgical pathology workflow that includes digital pathology will not reduce or remove the need to produce and ultimately store glass slides. Instead of any reductions, digital pathology will require additional workflows, personnel, equipment and importantly storage of data (it is estimated that digital pathology images constitute at least ten times larger files than radiology images) all on top of an already financially and operationally stressed health care system. Digital pathology will definitely bring some advantages especially in areas such as rapid teleconsultations with experts, quality and safety etc. Thus, proof of definite clinical value will be essential for widespread adoption of digital pathology. Given that digital pathology is likely to be costlier. AI in pathology will need to demonstrate improved efficiency, quality and safety [4, 6].

A major challenge to the deployment of digital pathology was recently addressed. In April 2019, Philips received FDA clearance for a Pathology Solution to be used for primary pathology diagnostics. This device is used for scanning glass pathology slides and for reviewing these slides on computer monitors. This Pathology Solution has already been established as a predicate device that could pave the way for a host of other FDA approved whole-slide scanners for primary diagnostics to become available in the coming years. Thus, the expanding role of AI in health care, the reduced costs of digital data and the availability of usable digital images are now in alignment for digital pathology to succeed [6].

Telepathology is defined as the practice of routine pathology using telecommunication links to enable the electronic transmission of digital pathology images. It can be used for remotely rendering primary diagnoses, second opinion consultations, quality assurance, education and research. Until recently the use of telepathology in clinical patient care was limited mostly to large academic institutions. In addition to prohibitive costs and legal and regulatory issues technological problems, lack of universal standards and most importantly resistance from pathologists slowed its widespread use. The adoption of telepathology practice is likely to expand in order the meet the increased demands for subspecialist consultation, and as technology advances, to improve diagnostic accuracy and work flow [29].

There are five main categories of digital or (tele) pathology i.e. static, dynamic, robotic, whole slide imaging (WSI) and hybrid methods. Telepathology systems from any of these categories can be used and have been found to provide timely and accurate diagnoses similar to conventional microscopy. It is important that these systems meet clinical needs and are validated for the intended use. The decision to purchase a particular system will depend on the clinical application, specific needs and budget of the laboratory as well as the personal preferences of the telepathologists involved [30].

In addition, the recent development of tissue clearing technology introduces the possibility of 3D pathology which allows for the collection of the 3D context of tissue and would contribute to increased accuracy of automatic pathological diagnosis by machine learning [31].

Automated analysis of histological slides is now possible through WSI scanners which can acquire and store slides in the form of digital images. This scanning associated with deep learning algorithms allows recognition of lesions through automatic recognition of regions of interest previously validated by the pathologist. These computers aided diagnostic techniques have already been tested in breast pathology and dermatopathology [32].

A 2016 study tested techniques for preprocessing of free-text breast cancer pathology reports with the aim of facilitating the extraction of information relevant to cancer morphology, grading and staging. These techniques included using freely available software to classify the reports as semi-structured or unstructured based on their general layout, and using an open source language engineering framework to predict parts of the report text which contained information relevant to the cancer. The results showed that it was possible to predict the layout of reports and that the accuracy of prediction as to which segments of a report contained relevant certain information was sensitive to the report layout and the type of information sought [33].

The digital revolution is transforming the practice of diagnostic surgical pathology by integrating image analysis and machine learning into routine surgical pathology. Thus a clear need is being felt for a robust and evidence based framework in which to develop these new tools in a collaborative manner that meet regulatory approval. A number of regulatory steps have been approved and implemented by the FDA in the United States and the NCRI Cellular Molecular Pathology (CM-Path) initiative and the British In Vitro Diagnostic Association (BIVDA) in the United Kingdom. These bodies have set out a road map to help academia, industry and clinicians develop new software tools to the point of approved clinical use. A 2019 study compared two commonly used CNNs in surgical pathology because it is often assumed that the quality and format of the training image as well as the number of training images in different convolutional networks impacts the accuracy of diagnosis. The authors photographed 30 hematoxylin and eosin (H&E) stained slides with normal tissue or carcinoma from breast, colon and prostate generating 3000 partially overlapping images (1000 per tissue type). They found that the images from the two convolutional networks were similar in their accuracy and large numbers of unique H&E stained slides were not required for training optimal ML models in diagnostic surgical pathology. The authors reinforced the need for an evidence based approach by comparing different ML models in order to achieve the best practices for histopathological ML ensuring an accurate diagnosis [34, 35]. Added value of quantitative AI in pathology includes the confirmation of equivocal findings noted by a pathologist, increasing sensitivity of feature detection, improving efficiency etc.

### Role of AI in surgical pathology in poor countries

Many authorities believe that there is no denying that quantitative AI is part of the future of pathology. However, the significant cost issues involved may be currently prohibitive for poor, developing countries. In these countries, the focus on ‘telepathology’ as a possible solution will be inadequate. Incorporating AI in telepathology can provide temporary solutions until requisite financing schemes are implemented. AI is especially applicable to surgical pathology because diagnosis depends on pattern recognition which is a useful quality for digital applications that depend on machine learning. AI has the potential to create online data repositories that can be used for the diagnosis of pathological specimens (e. g breast cancer) worldwide, greatly reducing human and infrastructural resource burden. A recent study showed that machine learning algorithms achieved potentially faster and more accurate diagnoses than 11 pathologists in a simulated setting. It is obvious that incorporation of AI in telepathology will demand high resources and entail heavy costs. These issues could be directly addressed if value is clearly demonstrated and results in government and third-party payers investing in reimbursement strategies for its use in pathology. The recognition of AI as part of reimbursement strategies that reward value-based care would provide important incentives to develop and implement validated algorithms. In developed western countries, the private sector is showing interest in investing in AI and other new technologies in healthcare. AI incorporation in pathology should be explored as a bold and creative idea in developing countries to motivate investment by private sector. The global efforts currently going on towards developing sustainable pathology and laboratory medicine services in low and middle income countries should include AI in pathology especially surgical pathology [29, 30, 34, 36].

However, other authorities disagree, at least in the context of surgical pathology. Some believe that although AI will play some role in diagnosis in the future, it could actually detract attention from proven, basic investments that are necessary to provide access to pathology and laboratory medicine services in low and middle income countries (LMICs). They argue that the use of AI in surgical pathology is still in its infancy and its ability to generate accurate diagnoses is yet to be proven. They believe that AI in telepathology will only be implemented in LMICs if it has first been successfully implemented in high income countries (HICs) where currently its role in day to day patient care remains unclear. These authors further argue that although telepathology could be used within integrated, tiered laboratory networks in LMICs, with slides prepared and scanned in lower levels and transferred to higher levels within the network for interpretation and consultation, telepathology relies on access to technology that is neither affordable nor practical in these countries. Most LMICs even lack the capacity to generate the slides needed as a prerequisite for telepathology and do not have access to these integrated networks. Even if it was affordable and possible to develop histopathology services, implement telepathology systems and transmit images taken in LMICs to pathologists in HICs as a temporary solution, there are insufficient numbers of pathologists in HICs to interpret images for large numbers of patients even in their own countries [37].

Will AI replace microscopes and pathologists? Notwithstanding the many difficulties and obstacles, it is now widely accepted that the use of AI will transform clinical practice over the next decade and some authors believe that an early impact of this will likely be the integration of image analysis and ML into routine surgical pathology. With a digital revolution transforming the reporting practice of diagnostic surgical pathology, a proliferation of image analysis software tools has resulted worldwide. This has ignited a hot debate among pathologists whether with increasing availability and refinement of image analysis software, surgical pathologists will ultimately be replaced by computer algorithms. In other words, will AI algorithms and computer programs replace pathologists and what therefore is the future of surgical pathologists? It is already widely accepted that AI algorithms “will be incredibly useful in medical research, diagnosis (and) complex treatment planning”. Currently, there appear to be many hurdles to replacing human microscopists with computer algorithms. On the practical side, as discussed previously there are significant financial barriers and costs to incorporating slide scanners and computers into pathology workflow, although presumably hospitals would undertake these steps if it was proved that computer algorithms improve diagnostic accuracy or increase the efficiency of pathologists [5, 38].

An especially important and interesting question is: will computer algorithms surpass humans in diagnostic abilities? Some authorities are skeptical and believe that notwithstanding the success of AI in radiology and cardiology, it is at present difficult to envision how AI can be integrated effectively into routine pathology practice. Pathology departments generate high resolution microscopy images which unlike radiology and cardiology do not correlate to equivalent standardized digital imaging formats and workflows. Images in pathology require a manual process of tissue biopsy, specimen preparation and staining before digitization. Currently, development of such state - of - the - art computer vision algorithms requires millions of training images. Although WSI which involves scanning the whole tissue on glass slides and digitizing the images is useful and allows many pathology slides to be analyzed efficiently within a relatively short period of time, the system nevertheless suffers from complications associated with acceptance, speed, the ability to digitize all types of tissues as well as issues of data resolution, storage and regulation. More importantly, establishing a whole slide image database of millions of images is currently not practical. Although there are research projects experimenting with digitized pathologic images, there is currently no standardized digital pathologic imaging workflow. Another problem is the size of data. It is currently impossible to directly feed whole slide images into algorithms because each one contains about 10GB of data. However newer studies have demonstrated a promising approach to circumvent this problem by dividing whole slide images into smaller patches and then training an algorithm to classify these patches into different categories. Once this is done, statistical summaries of patch diagnosis are fed into a machine learning algorithm to classify the entire image into a single diagnosis. Recent studies have shown that algorithms developed in this way are able to distinguish subtypes of non – small cell carcinoma of lung with an accuracy similar to that of expert pulmonary pathologists. In breast cancer, combining the predictions of human pathologists and algorithms led to an 85% decrease in human error in detecting metastatic breast cancer [5, 39–41].

In 2017, FDA approved the first WSI system which may encourage the pathology community to begin standardizing and using digitization on a larger scale thereby streamlining the exchange of information. The multidimensional nature of radiology or cardiology images allows them to be viewed on a 2 or 3 or 4 dimensional plane which provides rich information via AI pattern recognition. On the other hand, pathology images are digitized for 2 dimensional imaging of the sample which does not provide as much information as it would on a 3 or 4 dimensional plane. Currently, in the absence of standardized pathologic imaging workflow, the exchange and translation of information to other information systems, physicians and health systems is very difficult. Large centralized image archives of digitized pathologic images are not accessible compared to other medical imaging archives creating additional obstacles to the successful integration of AI into pathology practice. The development of such large centralized image archives or databases will require immense capital investment which may not be feasible for smaller hospitals. Thus even assuming that high speed AI algorithms can be developed to accurately detect and diagnose digitized pathology images, the gain in productivity from automation may be low in the face of the immense financial costs involved. One viable solution to this problem may be pathologic imaging management in the cloud computing environment provided adequate security privacy issue safeguards are ensured and the speed of image transfer over the internet is sufficient. If these are successful and a standardized digital imaging infrastructure in pathology is established, it will allow AI to become a powerful asset to pathologists who seek to better bridge the gap between research and patient care. In the near future, it is likely that technological advances like highly efficient automated whole slide scanner systems, innovative AI platforms, and pathologist friendly image annotation and analysis systems will become increasingly prominent in the daily professional lives of pathologists [5].

Many authors now predict that computers will become increasingly integrated into the pathology work flow and will especially be useful when they can improve accuracy in answering questions which are difficult for pathologists. It is predicted that computer programs i will be able to count mitotic figures or quantitatively grade immunohistochemistry stains more accurately than pathologists and could identify regions of interest in cytopathology slides thus reducing the time a pathologist would need to spend in screening. Some authors also predict that, over time, as computers gain more and more discriminatory abilities, they will reduce the amount of time it takes for pathologists to render diagnoses and in the process reduce the demand for pathologists as microscopists potentially enabling pathologists to focus their cognitive resources on higher level diagnostic and consultative tasks such as integrating molecular, morphologic and clinical information to assist in treatment and clinical management decisions for individual patients, in other words on personalized care. It is predicted that digital pathology, WSI and AI will be synergistic technologies to human cognition. The question of “human versus computer” is already being refined to “human versus human with computer”. It is believed that AI will enable pathologists to focus more on higher level cognitive tasks by performing the repetitive detailed tasks which require accuracy and speed and which humans find mind-numbing and consequently error prone. Pathologic diagnosis is considered to be a well-thought-out cognitive opinion, benefiting from the pathologists’ training and experience and subject to their biases. It is argued that the professional value of pathologists comes from their ability to give the most appropriate (even if not the most perfect) opinion in the clinical context and that human pathologists constantly recalibrate their diagnosis based on even small but significant bits of clinical and patient specific information provided through physician notes, pathology reports, verbal or written communications with clinicians etc. It is believed by many authors that a person working in partnership with an information resource is better than that same person unassisted. In other words, they favor “human versus human with computer” rather than “human versus computer”. They believe that a sunny era of AI assistance in pathology is on the horizon and do not believe in dark clouds of AI competition replacing pathologists. However, at the same time, they are realistic enough to recognize that eventually even the cognitive lead of human pathologists will narrow as new and better AI products emerge. Currently the whole frame work of AI, digital pathology and WSI depends on financial factors and remains undefined. It may, ironically, depend on human ability to overcome financial, technological and regulatory obstacles [42, 43].

Education of pathologists will be the greatest challenge and will require the longest times. AI methods will need to be integrated into all pathology training programs. Future generations of pathologists will need to be comfortable using digital images and other data in combination with computer algorithms in their daily practice. Optimistically, 5 to 10 years will be required to build such a work force even in developed countries and that too only if the process begins now. Many believe that AI may be just what pathology has been waiting for. While still requiring evaluation within a normal surgical pathology workflow, deep learning has the opportunity to assist pathologists by improving the efficiency of their work, standardizing quality and providing better prognostic information. Like in the case of immunohistochemistry and molecular diagnostics, there is little risk of pathologists being replaced. Although their workflow will likely change, their contribution to patient care will continue to be critically important. The diagnostic process is too complicated and diverse to be trusted to hard-wired algorithms alone. It is hoped that AI and human pathologists will be natural cooperators, not natural competitors. Thus, it appears AI will not replace the microscopist or take over pathology anytime soon. The hypothesis that intuition and creativity combined with the raw computing of AI heralds an age where well designed and executed AI algorithms will solve complex problems and replace the microscopist are not true. The microscope will probably be around for a long time. However, AI will likely play an increasingly important role in diagnostic microscopy. In the words of Granter et al., “winter may be coming but hopefully it will be gentle and mild.” [6, 38, 42].

### Current applications of AI in pathology

Let us now examine various examples of application of AI in practical surgical pathology especially in cancers and its impact on patient care.

As far back in 2007, Wild et al. used AI to predict the risk of progression to muscle invasion in non-muscle invasive bladder cancers. They integrated urinary bladder cancer arrays with artificial neural networks for this purpose. Although the recurrence rate of non-invasive bladder cancer is high, the majority of tumors are indolent and can be managed by endoscopic means alone. On the other hand, the prognosis of muscle invasion in bladder cancer is poor and radical treatment is required if cure is to be obtained. The authors developed a predictive panel of 11 genes to identify tumor progression. They found that the combination of genes analyzed using artificial neural networks was able to significantly stratify risk of tumor progression which was very difficult to identify by clinicopathological means [44].

In lung cancer, computational analysis of histological images using AI is now being increasingly applied in order to improve the diagnostic accuracy. Tumor phenotype usually reflects the overall effect of molecular alterations on the behavior of cancer cells and provides a practical visual reading of the aggressiveness of the tumor. However, in some cases, the human evaluation of histological images is subjective and lacks reproducibility. AI is now being increasingly used in routine clinical practice for the optimization of histological and cytological classification, prognostic prediction and genomic profiling of patients with lung cancer. However, there are still several challenges which need to be addressed for successful utilization of AI in the accurate diagnosis and prognostication of lung cancer [45].

Histopathological assessment of lung cancer slides is usually accurate in reaching a correct diagnosis. However, the prediction of prognosis is not very accurate. In a 2016 study, Yu et al. used fully automated microscopic pathology image factors to predict the prognosis of non-small cell lung cancer. They obtained 2186 H&E stained whole slide images of lung adenocarcinoma and squamous cell carcinoma patients from The Cancer Genome Atlas (TCGA) and an additional 294 histological images from Stanford Tissue Array (STA) database. From these images, they extracted 9879 quantitative image features and selected the top features by using regularized machine learning methods. They were able to distinguish short term survivors from long term survivors in patients with stage I adenocarcinoma and squamous cell carcinoma and showed that automatically derived image features can predict the prognosis of lung cancer patients accurately. Their findings were statistically significant. The authors are confident that their methods are extensible to histopathology images of cancers from other organs [39].

In a 2018 study, Kumar et al. built an automatic informatics methodology capable of identifying statistically significant associations between clinical findings of non-small cell lung cancers (NSCLC) in unstructured texts of patient pathology reports and the various clinically actionable genetic mutations identified from next generation sequencing (NGS) in NSCLC (EGFR, KRAS, BRAF and PIK3CA). Their findings were statistically significant (*p*-value < 0.05) and showed associations to mutations in specific genes which were consistent with published literature. Like Yu et al., Kumar et al. were also confident that their approach is extensible to other cancers and provide the first steps toward understanding the role of genetic mutations in the development and treatment of different types of cancer [40].

Considerable work utilizing AI has already been done in breast cancer pathology. In breast cancer, the most common malignancy in women worldwide, earlier diagnosis and better adjuvant therapy have substantially improved patient outcome in recent decades. Although pathological diagnosis has proved to be instrumental in guiding breast cancer treatment, new challenges have emerged as increased understanding of breast cancer in the last few years has revealed its complex nature. As patient demand for personalized breast cancer therapy grows, there is an urgent need for more precise biomarker assessment and more accurate histologic diagnosis to make better therapy decisions. The digitization of pathology data has opened the door to faster, more reproducible and more precise diagnosis through computerized image analysis. Software to assist diagnostic breast pathology through image processing techniques have been around for years. But recent breakthroughs in AI promise to fundamentally change the way breast cancer is detected and treated in the near future [46].

Nodal metastasis of breast cancer, or any other cancer for that matter, influences therapy decisions. Identification of tumor cells in lymph nodes can be laborious and error prone especially for small tumor foci. Steiner et al. developed a deep learning algorithm for the detection of breast cancer metastases in lymph nodes. In this study, six pathologists reviewed 70 digitized slides from lymph node sections unassisted and assisted by algorithms. In the assisted mode, the deep learning algorithm was used to identify and outline regions with high likelihood of containing tumor. Algorithm assisted pathologists demonstrated higher accuracy than either the algorithm or the pathologist alone. In particular, algorithm assistance significantly increased the sensitivity of detection for micro metastases (91% vs 83%, *p*-value = 0.02). Average review time per image was also significantly shorter with assistance than without assistance for both micro metastases and negative images (*p*-value = 0.002 and 0.018 respectively). The pathologists were asked to provide a numeric score regarding the difficulty of each image classification. On the basis of this score, pathologists considered the image review of micro metastases to be significantly easier when interpreted with assistance (*p*-value = 0.0005). This study demonstrates the potential of a deep learning algorithm to improve pathologist accuracy and efficiency in a digital pathology workflow [47].

In a 2017 study, Yala et al. developed a machine learning model to extract pertinent tumor characteristics from breast pathology reports which enabled them to create a large database. Their system was trained to extract 20 separate categories of information. They trained their system from two data sets which consisted of 6295 and 10,841 manually attenuated reports. It is important to note that extracting information manually from electronic medical records is a time-consuming and expensive process. The authors tested the accuracy of their model on 500 reports that did not overlap with the training set. The model achieved accuracy of 90% for correctly parsing all carcinoma and atypia categories for a given patient. The average accuracy for individual categories was 97%. Using this classifier, they created a database of 91,505 breast pathology reports from which information was extracted. They also developed a user – friendly interface to the data base that allows physicians to easily identify patients with target characteristics. The authors believe that their model has the potential to reduce the effort required for analyzing large amounts of data from medical records and to minimize the cost and time required to extract scientific information from these data [48].

In a 2018 study, Bychkov et al. developed and trained a deep learning network to predict outcome of colorectal cancer based on images of tumor tissue samples. They evaluated a set of digitized H&E stained tumor tissue microarray (TMA) samples from 420 colorectal cancer patients with known clinicopathological and outcome data. Their results showed that deep learning based outcome prediction with only small tissue areas as input outperformed visual histological assessment performed by human experts in stratification of patients into low risk and high risk categories. They suggested that state –of-the-art deep learning techniques can extract more prognostic information from the tissue morphology of colorectal cancer than an experienced human observer [49].

Yoshida et al. in a 2018 study evaluated the classification accuracy of a newly developed e – Pathologist image analysis software in gastric biopsies. They obtained and stained 3062 consecutive gastric biopsy specimens and digitalized the specimen slides. Two experienced gastrointestinal pathologists evaluated each slide for histological diagnosis. The authors compared the three tier (positive for carcinoma or suspicion of carcinoma; caution for adenoma or suspicion of a neoplastic lesion; or negative for a neoplastic lesion) or two – tier (negative or positive) classification results of human pathologists with the e-Pathologist. Overall concordance rate was 55.6%. For negative specimens, concordance rate was 90.6% but for positive biopsy specimens, concordance rate was less than 50%. For the two tier classification, sensitivity and specificity were 89.5 and 50.7% respectively. The authors concluded that although there are limitations to the application of automated histopathological classification of gastric biopsy specimens in the clinical setting, the results show promise for the future [50].

The shortage of well – annotated pathology image data for training deep neural networks is currently a major issue because of the high costs involved. To overcome this, transfer learning techniques are generally used to reinforce the capacity of deep neural networks. In order to further boost the performance of the state-of-the-art deep neural networks and overcome the insufficiency of well annotated data, Qu et al. presented a novel stepwise fine tuning-based deep learning scheme for gastric pathology image classification. Their proposed scheme proved capable of making the deep neural network imitating the pathologist’s professional observations and of acquiring pathology – related knowledge in advance but with very limited extra cost in data annotation. They conducted their experiments with both well-annotated gastric pathology data and the proposed target-correlative intermediate data on several state-of-the art deep neural networks. Their results demonstrated the feasibility and superiority of their proposed scheme for boosting the classification performance [51].

A recent study by Liu et al. evaluated the application and clinical implementation of a state-of-the-art deep-learning-based AI algorithm. (Lymph Node Assistant or LYNA) for detection of metastatic breast cancer in sentinel lymph node biopsies. They obtained whole slide images from H&E stained lymph nodes from 399 patients (publicly available Camelyon 16 challenge data set). LYNA was developed by using 270 slides and evaluated on the remaining 129 slides. The findings were compared with 108 slides (86 blocks) from 20 patients obtained from an independent laboratory using a different scanner to measure reproducibility. LYNA achieved a slide-level area under the receiver operating characteristic (AUC) of 99% and a tumor level sensitivity of 91%. It was not affected by common histological artifacts such as poor staining, air bubbles or over fixation. The AI algorithm exhaustively evaluated every tissue patch on a slide and achieved higher tumor-level sensitivity than, and comparable slide-level performance to, pathologists. This study once again showed that AI algorithms may improve the pathologist’s productivity and reduce the number of false negatives associated with morphologic detection of tumor cells. The authors provide a frame work to aid practicing pathologists in assessing AI algorithms for adoption into their workflow (akin to how a pathologist assesses immunohistochemistry results) [52].

An International contest was held recently to have a machine detect sentinel lymph node metastases of breast cancer. It was termed the CAMELYON 16 grand challenge (Cancer Metastases in Lymph Nodes Challenge) and different teams submitted deep learning algorithms for this purpose. The findings of AI algorithms were compared with those of a panel of 11 pathologists with varying degrees of expertise in breast pathology. Although the top algorithms performed better than the 11 pathologists when they were under time constraint, they did not perform differently from the pathologists when the latter had unlimited time. However, the fact that the algorithms detected nodal micro metastases at the same rate or better than pathologists was exciting. The fact that pathologists were able to do as well or even better than the algorithms when there were no time constraints is significant. The CAMELYON 16 challenge highlights a significant opportunity for AI in pathology, namely assisting pathologists with screening for lesions [53].

In surgery for laryngeal carcinoma, preservation of adjacent healthy tissue is very important. Thus, accurate and rapid intraoperative histology of laryngeal tissue is critical to achieve optimal surgical outcomes. Zhang et al. used deep-learning based Stimulating Raman Scattering (SRS) microscopy to provide accurate automated diagnosis of laryngeal squamous cell carcinoma on fresh surgical specimens without fixation, sectioning, staining or processing. The authors demonstrated near perfect concordance between SRS and standard histology evaluated by pathologists. Their deep learning based SRS classified 33 surgical specimens with 100% accuracy. The authors contended that SRS histology integrated with deep learning algorithms has the potential for providing rapid intraoperative diagnosis which could help in the surgical management of laryngeal carcinoma [54].

Yadav et al. recently showed how AI is optimizing the detection and management of prostate cancer through integration of machine learning based identification of Gleason scores from pathology slides with genomics, imaging (especially MRI) and biomarkers [55].

It is predicted that the laboratory of the future will be more highly automated and dominated by robotics and will be more connected to take advantage of the benefits of AI and the Internet of Things [56].

## Conclusion

AI, the theory and development of computer systems which can perform tasks which normally require human intelligence, is slowly becoming part of everyday modern life. Health care was slow to embrace AI but the pace of implementation has now picked up. Computer based decision support systems based on Machine Learning can perform complex tasks which are currently assigned to specialists. This has the potential to revolutionize medicine by increasing diagnostic accuracy, improving clinical workflow, decreasing costs of human resources and improving therapeutics. Growing interest in AI and machine learning in diverse industries including health care is mainly due to the rise of Deep Learning, a process through which AI recognizes patterns using various forms of neural networks which resemble the human brain and which are in turn based on the availability of big data repositories. The promise of AI in health care is to deliver improved quality and safety of care and to democratize expertise through use of mobile devices such as smart phones which can be deployed with algorithms and potentially be accessible universally at low cost anywhere in the world delivering vital diagnostic care. Health care is ripe for AI because it has rich data sets (big data) which are ideal for AI since computers require large data sets to learn. AI is thus fast becoming a major element in the health care landscape. AI algorithms will in the coming future play an important role in predicting cancer outcome and assisting in therapeutic decisions for cancer patients. In fields like radiology which has already embraced digital operations, an AI revolution is already in progress. Deep neural networks will be able to provide a synergistic combination of disciplines such as radiology, nuclear medicine and surgical pathology which will hopefully allow the achievement of a medical paradigm which recognizes that every human being is unique. Although pathology especially surgical pathology was late to adopt AI, mainly due to practical and financial obstacles, and will require resources for additional workflows, personnel, equipment, storage of data, the time is now ripe, with rapid development of new and better AI technology at lower cost (reduced costs of digital data and availability of digital images) for AI to succeed in surgical pathology. Various studies cited above demonstrate the increasingly effective role of AI in surgical pathology. By increasing speed and accuracy of diagnosis and by improving prognostication, use of AI is translating into better patient care. AI will, in the near future, not replace pathologists but by performing routine repetitive tasks quickly and accurately, allow pathologists to give time to more complex cognitive tasks and enable them to play a much greater and effective role in cancer prognosis and therapeutics. Thus pathologists need to embrace AI and derive benefit from it by training themselves. This will require considerable time as AI methods will need to be integrated into pathology training programs and pathologists will need to be comfortable using digital images and data with computer algorithms in their daily practice. With regulatory control also being established by government agencies in many countries such as US and UK, the reliability of AI and the trust of the general public will increase substantially and help in better care of patients which is the prime purpose of all efforts to improve medical technology. In this context, the synergistic collaboration between fields such as oncology, radio diagnostics and surgical pathology will play a major role. Financial barriers will need to be overcome especially for poor developing countries so that they can also benefit from improvements in AI application in medicine and pathology.

## Data Availability

Data and materials of this work are available from the corresponding author on reasonable request.

## Abbreviations

AI: Artificial Intelligence
ML: Machine Learning
CNNs: Convoluted neural networks
IoT: Internet of Things
SCC: Squamous Cell Carcinoma,
ICU: Intensive care unit
OR: Operating room
CAD: Computer aided diagnosis
MRI: Magnetic Resonance Imaging
CT: Computed Tomography
WFO: Watson for oncology
WSI: Whole slide imaging
FDA: Food and Drug Administration
CM-Path: Cellular Molecular Pathology
BIVDA: British In Vitro Diagnostic Association
H&E: Hematoxylin and Eosin
LMIC: Low and middle income country
HIC: High income country
TCGA: The Cancer Genome Atlas
STA: Stanford Tissue Array
NSCLC: Non-small cell lung cancer
NGS: Next generation sequencing
TMA: Tissue microarray
LYNA: Lymph node assistant
SRS: Stimulating Raman Scattering.
